# Platelet-Rich Plasma (PRP) Rinses for the Treatment of Non-Responding Oral Lichen Planus: A Case Report

**DOI:** 10.3390/biomedicines6010015

**Published:** 2018-02-06

**Authors:** Elisabetta Merigo, Aldo Oppici, Anna Parlatore, Luigi Cella, Fabio Clini, Matteo Fontana, Carlo Fornaini

**Affiliations:** 1 Dentistry, Special Needs and Maxillo-Facial Surgery Unit, Hospital “Guglielmo da Saliceto”, 29100 Piacenza, Italy; a.oppici@ausl.pc.it (A.O.); l.cella@ausl.pc.it (L.C.); f.clini@ausl.pc.it (F.C.); m.fontana@ausl.pc.it (M.F.); carlo@fornainident.it (C.F.); 2 Micoralis Laboratory EA7354, University of Nice “Sophia Antipolis”, 24 Avenue des Diables Bleus, 06100 Nice, France; 3 Tranfusional Unit, Hospital “Guglielmo da Saliceto”, 29100 Piacenza, Italy; a.parlatore@ausl.pc.it

**Keywords:** Oral Lichen Planus, platelet-rich plasma (PRP), wound healing

## Abstract

Platelet-rich plasma (PRP) has been proposed for different applications in the medical field and in maxillofacial surgery thanks to its many growth factors, such as epidermal growth factor (EGF), fibroblast growth factor (FGF), and keratinocyte growth factor (KGF). Oral lichen planus (OLP) is a disease that affects the oral mucosa in a chronic way. This disease frequently worsens the quality of life of patients, particularly when clinical manifestations are of the erythematous or erosive/ulcerative type. The properties of PRP that are supported by scientific literature in both oral medicine and other medical fields have suggested the introduction of PRP in clinical practice for the medical treatment of different soft tissues diseases, such as when OLP patients do not respond to conventional therapies, or when conventional treatments have some contraindications or side effects. The aim of this work is to describe the use of PRP used as an oral rinse for the treatment of a patient diagnosed as affected by OLP at the Dentistry, Special Needs and Maxillo-Facial Surgery Unit of the Hospital of Piacenza. PRP protocol was started after the failure of conventional therapies based on the use of topical and systemic corticosteroids, hydroxychloroquine, and low-level laser therapy applications.

## 1. Introduction

Oral lichen planus (OLP) is a disease that affects the oral mucosa in a chronic way that frequently worsens the quality of life of patients, particularly when clinical manifestations are of the erythematous or erosive/ulcerative type [1].

Totally curative treatment is difficult to find and, even if topical corticosteroids as a first choice treatment allow good results, the side effects and not responding cases force the discovery of other kinds of therapies [1].

Actually, targeted therapies as biologic agents or natural agents such as curcumin, aloe vera, and vitamin A are proposed, but none of them has been estimated as superior to topical steroids as a first-line therapy for the management of OLP [2].

Also, laser utilization was proposed for both surgery and biomodulation, particularly to reduce the OLP symptoms, and to achieve a better quality of life for patients [3,4].

Autologous platelet-rich plasma (PRP) is a concentration of human platelets that is three to five times greater than the physiologic concentration of thrombocytes in whole blood; this product is characterized by large amounts of growth factors, which are released after platelet activation and are able to stimulate the production of collagen and extracellular matrix. 

Specifically, PRP contains platelet-derived growth factors (PDGF) and transforming growth factor (TGF)-β, which are able to stimulate the proliferation of mesenchymal cells; it also contains vascular endothelial growth factors (VEGFs) and fibroblast growth factor (FGF), which are able to stimulate new blood vessel formation [5]. This is the reason why the usage of platelet-rich plasma has been introduced as an adjunctive therapy in different medical fields [6].

It is reported that the total amount of growth factors is released around one hour after platelet activation, while 70% of the growth factors are released 10 min after the activation of thrombocytes [7]; the autologous use is free from immune reaction or allergy, and severe side effects are rare in the literature [8].

In oral medicine and maxillofacial surgery, PRP use has been described, for example, for the treatment of bone diseases such as medication-related osteonecrosis of the jaws (MRONJ), particularly in addition to surgical protocol as a gel for topic application [9,10].

In ophthalmology, PRP has been used as eye drops for the treatment of dry eye, and for ocular lesions as corneal erosions [11].

## 2. Description of the Case

**Figure 1:**
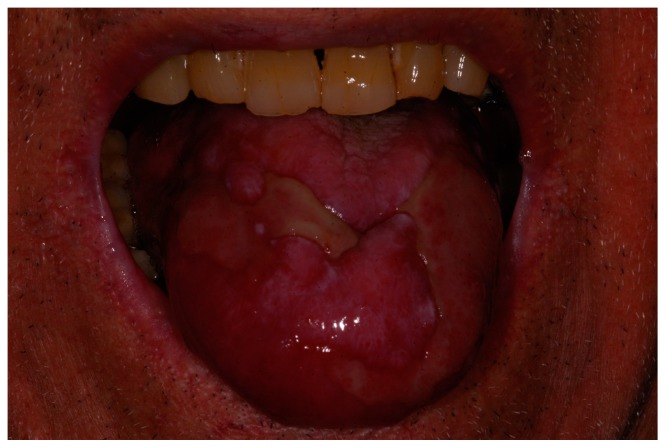
Clinical view of the patient at the first evaluation: tongue ulcerative lesions were making it impossible for the patient feeding with solid food.

A 73-year-old man with positive anamnesis for hypertension and treatment with acetylsalicyclic acid presented at the Dentistry, Special Needs and Maxillo-Facial Surgery Unit of the Hospital of Piacenza with large ulcerative lesions on his tongue impeding to him a normal and painless food intake (Figure 1). Blood examination was negative for alterations on blood cell count, hepatic parameters (Glutamic Oxaloacetic Transaminase or GOT, Glutamic Pyruvic Transaminase or GPT, bilirubin) and antitumoral markers (test for carcinoembryonic antigen or CEA, Cancer Antigen 19-9, or CA 19-9) and positive for Hepatitis C Virus (HCV). Incisional biopsy was taken on the tongue, and the result was positive for oral lichen planus. In the days before biopsy, the patient was treated with topical hyaluronic acid gel three to four times a day without any improvement.

Topical treatment with clobetasol 0.05% ointment mixed in equal parts (50:50) with hydroxypropyl cellulose 5%, was performed three times a day with prophylactic treatment with nystatin rinses three times a day for five weeks without any improvement, nor was there any improvement with the adding of betamethasone 0.5 mg for oral rinses three times a day.

Due to the poor quality of life of the patient, treatment with systemic corticosteroid was started with prednisone 0.5 mg/kg once a day for oral administration, and azathioprine 50 mg once a day for oral administration for two weeks, but this was stopped because of the increase of blood tension.

Daily treatment with 200 to 400 mg hydroxychloroquine sulfate, an antimalarial agent, was administered as a monotherapy for three months without any clinical or symptomatological improvement.

**Figure 2:**
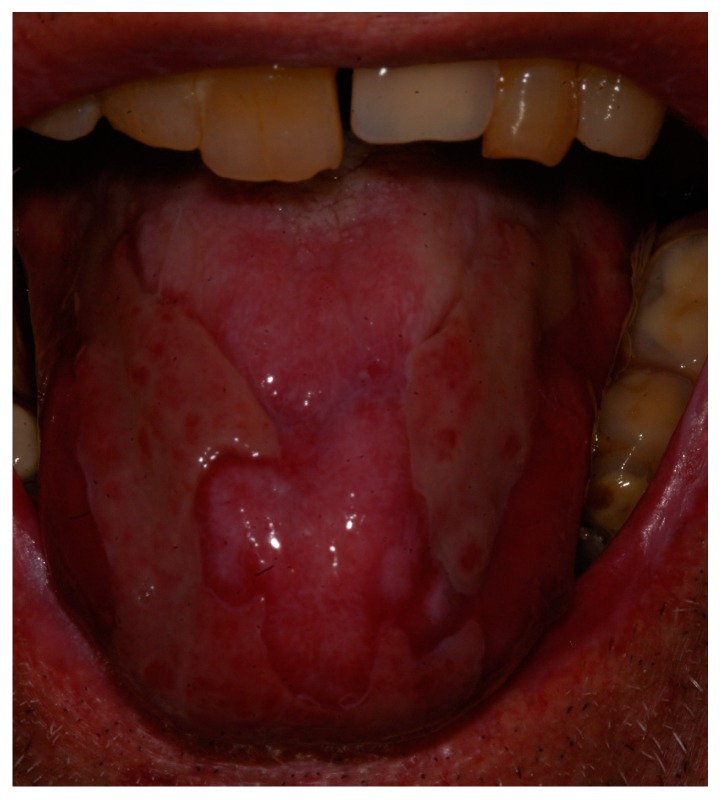
Clinical view of the patient after one year of conventional therapies.

Laser biomodulation protocol was then performed with a 808-nm diode laser with a 600-μm diameter fiber on a scanning mode on the tongue lesions sites with 1 W of power in continuous and non-contact mode for 1 min five times (Theorical fluence: 21,231 J/cm^2^); treatment was performed twice a week for a month: the patient described a symptomatological improvement, allowing more comfort during meals, but tongue lesions did not have a significant modification (Figure 2).

Autologous platelet solution was then prepared at the Department of Oncology and Hematology of the Hospital of Piacenza (Italy): multiple samples of whole blood of the patient himself were taken and collected in 10-mL Vacutainers after the patient signed an informed consent agreement. Blood was then centrifuged at 180 rpm for 10 min in order to separate the concentrated erythrocytes from platelet-rich plasma (PRP). This PRP preparation was stored at −40 °C; then, daily doses were prepared and given to the patient for a whole month of treatment with the indications to store the bottles at −20 °C until use, and to maintain the bottles being used under refrigerated conditions at 4 °C. 

**Figure 3:**
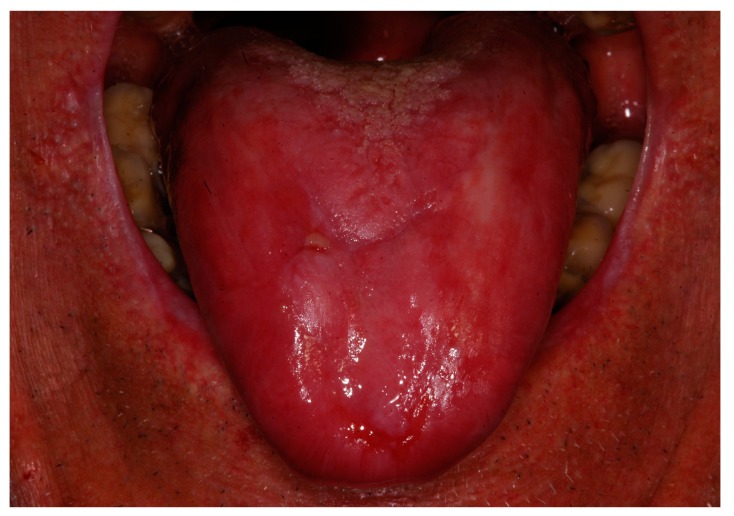
Clinical view of the patient after two months of treatment with platelet-rich plasma (PRP) oral rinses.

The patient was followed every two weeks and at every clinical examination he reported improved comfort, with a decrease of pain, spontaneous or provoked by food, together with an objective clinical improvement with a resolution of ulcerative lesions after two months of therapy (Figure 3).

## 3. Discussion and Conclusions

The growing use of blood-derived products is directly linked to their combination of efficiency and safety for different wound-healing processes. In a systematic review, Martínez-Zapata et al. [12] described the efficacy of autologous PRP in randomized controlled trials (RCTs) on oral and maxillofacial applications, chronic skin ulcers, and wound healing after surgery; the same authors analyzed the safety profile for PRP concluding that, even if a bias exists through small sample sizes and lower quality studies, there is no evidence for any relation between PRP and adverse events. 

The case we presented is an example of the potential for this kind of treatment in cases where conventional therapy is not able to heal the wound, or where side effects are too important and dangerous for the patient.

Obviously, there is a great need for more detailed prospective studies on a larger group of patients with a longer follow-up period.
